# *Plasmodium berghei* Calcium Dependent Protein Kinase 1 Is Not Required for Host Cell Invasion

**DOI:** 10.1371/journal.pone.0079171

**Published:** 2013-11-12

**Authors:** Sylvia Jebiwott, Kavitha Govindaswamy, Amos Mbugua, Purnima Bhanot

**Affiliations:** Department of Microbiology and Molecular Genetics, Rutgers New Jersey Medical School, Rutgers, The State University of New Jersey, Newark, New Jersey, United States of America; Tulane University, United States of America

## Abstract

*Plasmodium* Calcium Dependent Protein Kinase (CDPK1) is required for the development of sexual stages in the mosquito. In addition, it is proposed to play an essential role in the parasite’s invasive stages possibly through the regulation of the actinomyosin motor and micronemal secretion. We demonstrate that *Plasmodium berghei* CDPK1 is dispensable in the parasite’s erythrocytic and pre-erythrocytic stages. We successfully disrupted *P. berghei* CDPK1 (PbCDPK1) by homologous recombination. The recovery of erythrocytic stage parasites lacking PbCDPK1 (PbCDPK1-) demonstrated that PbCDPK1 is not essential for erythrocytic invasion or intra-erythrocytic development. To study PbCDPK1’s role in sporozoites and liver stage parasites, we generated a conditional mutant (CDPK1 cKO). Phenotypic characterization of CDPK1 cKO sporozoites demonstrated that CDPK1 is redundant or dispensable for the invasion of mammalian hepatocytes, the egress of parasites from infected hepatocytes and through the subsequent erythrocytic cycle. We conclude that *P. berghei* CDPK1 plays an essential role only in the mosquito sexual stages.

## Introduction

Ca2+ signaling plays a crucial role in Apicomplexan parasites. It mediates micronemal protein secretion in *Plasmodium* [1], *Toxoplasma* [2]–[4], *Cryptosporodium* [5] and *Eimeria* [6]. In *Plasmodium*, Ca2+ is a key mediator of egress from the infected erythrocyte [7]–[10], gametogenesis and ookinete motility in the mosquito [11]–[13]. It is also implicated in sporozoite invasion of hepatocytes [14].

A major mediator of Ca2+ signaling in Apicomplexans is a family of Calcium Dependent Protein Kinases (CDPK) that is unique to Apicomplexans, plants and some algae. Apicomplexans express five major classes of CDPKs all of which contain Ca2+-binding EF-hand motifs linked to a kinase domain [15]. *P. falciparum* has seven CDPK homologs belonging to four classes. Plasmodium CDPK1 is conserved in all *Plasmodium* species and has homologs in *Toxoplasma gondii* and *Cryptosporidium parvum* [15]. It is expressed through out the parasite lifecycle [16], [17] suggesting that it has multiple roles in different parasite stages. It is best studied in *Plasmodium berghei* sexual stages, where CDPK1 controls the transcription of a subset of translationally-repressed mRNAs, and a knock down of *P. berghei* CDPK1 (PbCDPK1) blocks ookinete development [16]. In asexual stages, CDPK1 is implicated in parasite invasion based on three lines of evidence. First, *Plasmodium falciparum* CDPK1 (PfCDPK1) is transcriptionally coexpressed with components of the parasite’s actinomyosin motility apparatus and can phosphorylate key components such as the glideosome associated protein 45 (GAP45) and the myosin tail-interacting protein (MTIP) *in vitro* [17]–[19]. Second, small molecule and peptide inhibitors of PfCDPK1 block *P. falciparum* schizogony [17] and micronemal secretion [20], respectively. Third, attempts to obtain *P. falciparum* and *P. berghei* parasites with disrupted CDPK1 have failed (Kato et al, 2008; [16], suggesting that CDPK1 is essential for the parasite’s erythrocytic cycle. CDPK1’s role in sporozoites is yet to be determined. Here we report a comprehensive genetic strategy in *P. berghei* to examine CDPK1’s function throughout *Plasmodium’s* lifecycle.

## Results and Discussion

### CDPK1 is Dispensable for the Erythrocytic Cycle

**Figure 1:**
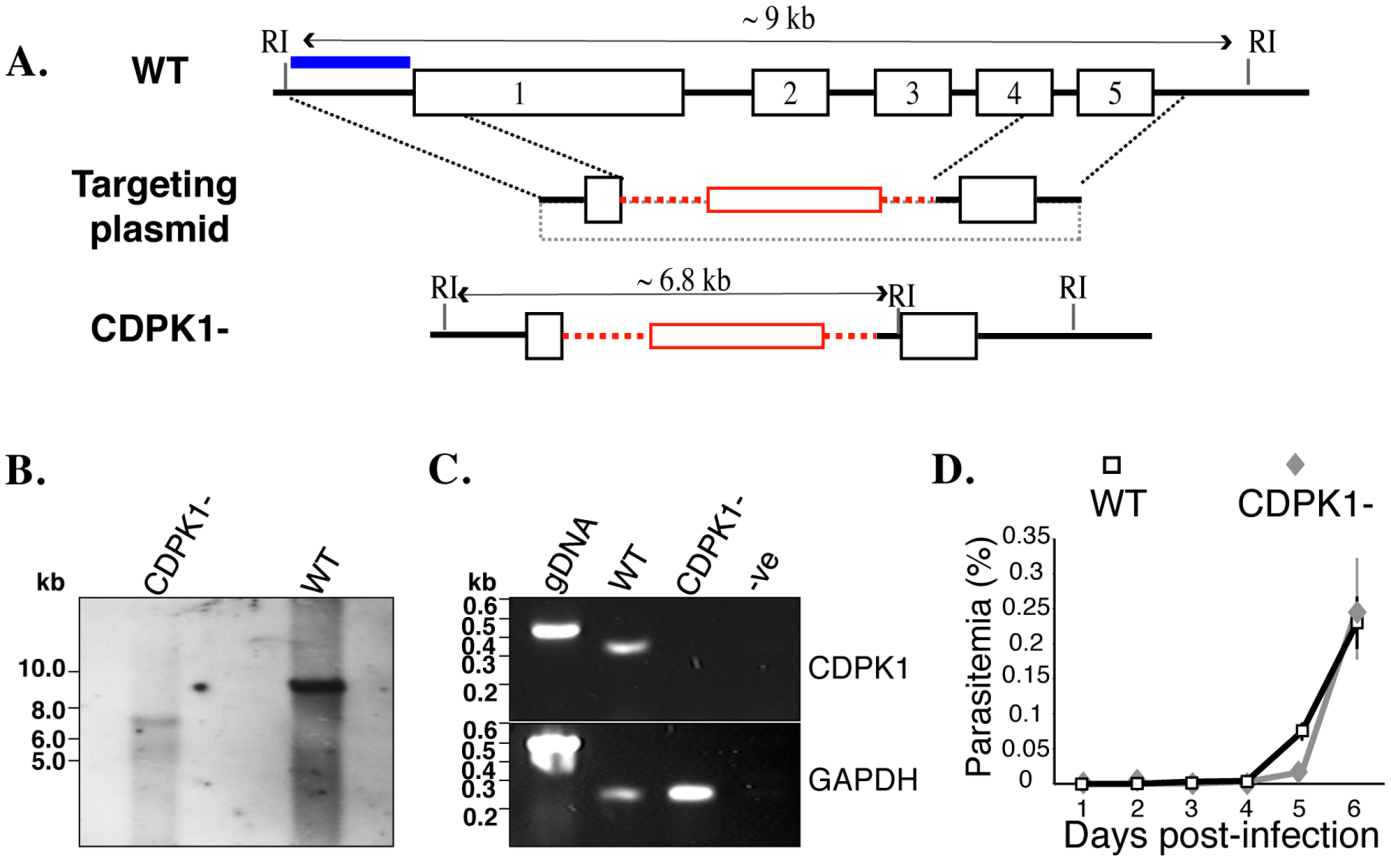
CDPK1- parasites are viable during erythrocytic development. (A) Schematic of PbCDPK1 knockout strategy using homologous recombination. (B) Southern hybridization, using a probe indicated in blue, demonstrates replacement of the PbCDPK1 locus. (C) RT-PCR demonstrates the loss of PbCDPK1 expression in CDPK1- parasites. PbCDPK1 and GAPDH control transcripts were amplified from genomic DNA from WT parasites, from cDNA of WT erythrocytic stages, from cDNA of CDPK1- erythrocytic stages or from water as negative control. (D) CDPK1- erythrocytic stage parasites have a growth rate similar to wildtype *in vivo*, as determined by daily monitoring of parasitemia of infected mice.

In order to test CDPK1’s function in the erythrocytic cycle, we attempted to generate a direct knockout of *P. berghei* CDPK1 (PbCDPK1) (Fig. 1A). Contrary to previous reports [21], we were successful in recovering the knockout parasites (CDPK1-) (Fig. 1B). We confirmed the loss of CDPK1 expression during erythrocytic development in CDPK1- mutant parasites using RT-PCR (Fig. 1C). The recovery of parasites lacking CDPK1 demonstrates that PbCDPK1 is not essential during the erythrocytic cycle. To determine if lack of PbCDPK1 compromises intra-erythrocytic development in the parasite, we monitored the growth rate of CDPK1- erythrocytic stages in mice (Fig. 1D). The parasitemia of CDPK1- and wildtype (WT) parasites was similar, suggesting that CDPK1- parasites do not suffer from a significant growth deficit. Therefore, PbCDPK1 function is either redundant or dispensable during erythrocytic invasion, intracellular development and egress. Previously reported failures to obtain CDPK1- mutants may be attributed to technical differences.

A sexual-stage specific knockdown of PbCDPK1 inhibits ookinete development [16]. PbCDPK1 is required for the translational activation of mRNAs in the developing zygote [16]. Consistent with these previous reports, CDPK1- parasites did not form oocysts in the mosquito midgut. The average number of oocysts in the midguts of WT-infected mosquitoes was 37+9 (n = 8) and in CDPK1- infected mosquitoes was 0 (n = 8).

### CDPK1 is not Essential in Pre-erythrocytic Stages

PbCDPK1 and one of its putative substrates, MTIP are present in sporozoites [16]. We hypothesized that PbCDPK1 may function in sporozoite invasion of hepatocytes. Since CDPK1- parasites do not complete sexual development in the mosquito, studying the function of PbCDPK1 in pre-erythrocytic stages required a conditional mutagenesis approach.

**Figure 2:**
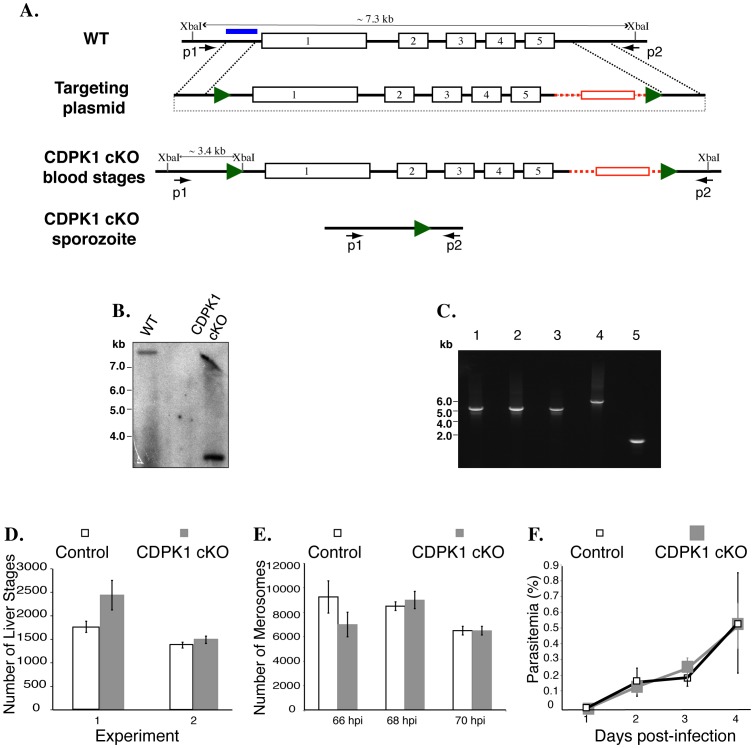
Conditional mutagenesis of PbCDPK1. (A) Schematic representation of the conditional mutagenesis of the PbCDPK1. (B) Southern hybridization demonstrating modification of the PbCDPK1 locus through the addition of FRT sites in FlpL/TRAP parasites. (C) PCR analysis using oligos p1 and p2, demonstrates excision of PbCDPK1 in genomic DNA obtained from (1) WT erythrocytic stages (2) FlpL/TRAP erythrocytic stages used for mosquito feeding (3) FlpL/TRAP erythrocytic stages recovered through bite infection from infected mosquitoes (4) CDPK1 cKO erythrocytic stages used for mosquito feeding (5) CDPK1 cKO erythrocytic stages recovered through bite infection from infected mosquitoes. (D) Intrahepatic development of CDPK1 cKO and control FlpL/TRAP sporozoites in HepG2 cells. Intracellular liver stages were quantified at 40–44 h p.i. (D) Parasite egress from infected HepG2 cells was monitored by quantifying the number of extracellular merosomes 66–70 h p.i. (E) *In vivo* infection of CDPK1 cKO and control FlpL/TRAP sporozoites was monitored by the appearance of erythrocytic stage parasites in mice infected through sporozoite injection.

We generated conditional mutants (CDPK1 cKO) in which oocyst formation and sporozoite development is normal. We used the Flp-FRT system [22] to bypass the requirement for PbCDPK1 in the parasite’s sexual cycle. The PbCDPK1 open reading frame was modified by the addition of flanking FRT sites in parasites expressing FlpL recombinase under the control of the TRAP promoter (FlpL/TRAP) [22] (Fig. 2A, B). In this system, PbCDPK1 is expressed normally during erythrocytic development and the sexual cycle in the mosquito. However, the open reading frame is excised during sporozoite development in the mosquito midgut generating mature sporozoites that lack PbCDPK1 (CDPK1 cKO) (Fig. 2C).

Equal numbers of sporozoites were recovered from salivary glands of CDPK1 cKO-infected and FlpL/TRAP-infected mosquitoes, demonstrating that CDPK1 is not required for parasite invasion of salivary glands. To determine if CDPK1 plays a role in hepatocyte invasion, we used CDPK1 cKO sporozoites to infect the human hepatoma cell line, HepG2. FlpL/TRAP sporozoites were used as controls in this and subsequent experiments. There was no significant difference in the number of liver stages formed by CDPK1 cKO or control sporozoites (Fig. 2D). These results demonstrate that CDPK1 is not essential for the parasite’s invasion of hepatocytes or subsequent intrahepatic development. To study CDPK1’s role in parasite egress from infected HepG2 cells, we determined the number of extracellular merosomes released in CDPK1 cKO and control infected HepG2 cultures. There was no significant difference in the number of merosomes present in both cultures (Fig. 2E). These results indicate that CDPK1 is not essential for parasite development in and egress from hepatocytes.

To determine if CDPK1 cKO sporozoites infect normally *in vivo*, we infected mice with either CDPK1 cKO or control sporozoites and monitored the appearance of erythrocytic stage parasites. Mice infected with CDPK1 cKO sporozoites had a normal pre-patent period of infection and there was no significant difference in the growth of erythrocytic stage parasites (Fig. 2F).

Our results demonstrate that despite being expressed throughout the parasite life-cycle, PbCDPK1’s essential role is in ookinete development. During invasion and egress by asexual stages and sporozoites, PbCDPK1’s function appears to be redundant. Since *P. berghei* has 6 CDPK homologs and multiple CDPKs are expressed in the same parasitic stage, it is likely that their functions are overlapping. This model is supported by the limited efficacy in *P. falciparum* cultures of purfalcamine, a potent *in vitro* inhibitor of recombinant PfCDPK1 [17]. Our results suggest that the purfalcamine’s low efficacy in *P. falciparum* cultures may be attributed to PfCDPK1 being non-essential in erythrocytic stage parasites rather than poor pharmacokinetic properties. Failure to disrupt PfCDPK1 may result from a low rate of recombination at the PfCDPK1 locus and may not necessarily reflect an essential function for PfCDPK1. Indeed a specific conditional knock-down PfCDPK1 in erythrocytic stages achieved a very significant decrease in protein levels but did not reveal a defect in intraerythrocytic growth [23].

An alternative model to explain our results is that CDPK1 has an essential role in erythrocytic stage invasion of *P. falciparum* but not of *P. berghei*., However, the conservation of the motor components between *P. berghei* and P. falciparum suggests that their regulatory mechanism will be similarly conserved. The *Toxoplasma gondii* ortholog of CDPK1 (TgCDPK3) is also dispensable for tachyzoite invasion and motility [24]–[26]. TgCDPK3 knockout tachyzoites are comparable to wild-type in normal egress. They demonstrate a delay only in ionophore-induced egress. This TgCDPK3-dependent mechanism of tachyzoite egress revealed by ionophore treatment is likely to be limited to a subset of intracellular conditions encountered by *T. gondii* in the host. Support for PfCDPK1’s essential role during the erythrocytic cycle was provided by the developmental arrest in early schizogony triggered by the ectopic expression of PfCDPK1’s auto-inhibitory junction domain in *P. falciparum* [23]. However, the interpretation of these results is confounded by the potential for non-specific effects. For example, the ectopically expressed junction domain may bind and inhibit related kinases required during intra-erythrocytic development. Future work will focus on distinguishing between the two proposed models using additional genetic tools. Our work reveals redundancy in the *Plasmodium* kinome and will influence future attempts to develop drugs that target multiple CDPK homologs to increase their efficacy.

## Materials and Methods

### Ethics Statement

This project was carried out in accordance with the recommendations of the Guide for the Care and Use of Laboratory Animals of the National Institutes of Health. The animal protocol was approved by the Animal Care and Use Committee of the New Jersey Medical School (protocol number P132D1113).

### Generation of CDPK1- Parasites

The CDPK1- targeting plasmid was constructed in pL0001. A 0.6 kB fragment encompassing the 5′ UTR region of PbCDPK1 was amplified using primers 5′GGTACCGCTTTTACCTGGCAAAAGT and 5′CCTGCAGGCTTTTACTTT-GATTACACCC. The product was cloned into the pL0001 vector using KpnI an SbfI (underlined). Another 0.6 kB fragment encompassing exon 5 and the 3′UTR of PbCDPK1 was amplified using primers 5′GGATCCTTCACTTTTTTTCATTTGTTTTT and 5′ACTAGTTAAACCTGTCTTTT-TTTTTATG. The product was cloned into pL0001 containing the 5′ PCR product using BamHI and SpeI (underlined). The final insert was released from the plasmid using KpnI and SpeI. P. berghei ANKA schizonts were transfected following standard methodology. Transfectants were selected by pyrimethamine treatment and cloned by limiting dilution.

### Generation of CDPK1 cKO Parasites

The cKO targeting vector was constructed by cloning three PCR products into a vector containing FRT sites and the human dihydrofolate reductase expression cassette [22]. A 1.0 kB fragment encompassing the 5′ UTR region of PbCDPK1 was amplified using primers 5′GCGGCCGCGAGATGTTTTGCATAGTA and 5′GCGGCCGCTATTTTTTTTATTA-TTTTTTATTA. The product was cloned into the vector NotI (underlined). A 3.0 kB fragment encompassing exons 1–5 of PbCDPK1 was amplified using primers 5′CTCGAGTGAACATGTGCATGTATA and 5′CTCGAGTATTTAAGCGCTTACCATTA and inserted into the previously generated plasmid using XhoI (underlined). A 0.5 kB fragment was amplified using primers 5′GGTACCCATAAAAAAAAAGACAGG and 5′GAATTCAAAAAAGGGGAGCAGGG and inserted into the previously derived plasmid using KpnI (underlined) and EcoRI (underlined). The final insert was released from the targeting construct using NotI and EcoRI. Transfections of the targeting plasmid into PbA TRAP/FlpL parasites [22] were carried out using standard methodology. Transfected parasites were selected using pyrimethamine and cloned by limiting dilution. Parasites were passaged into *Anopheles stephensi* mosquitoes.

### Mosquito Cycles

*Anopheles stephensi* mosquitoes were fed on CDPK1- infected or CDPK1cKO-infected Swiss-webster mice. CDPK1 cKO-infected mosquitoes were maintained at 20°C until day 14 post bloodmeal and then transferred to 25°C. PbA TRAP/FlpL parasites were used as controls. Sporozoites were dissected from their salivary glands at days 18–21 post feeding. Infectivity of salivary glands was similar for cKO and control parasites at approximately 10000–15,000 sporozoites/mosquito. Midguts were dissected from CDPK1- infected mosquitoes at day 9 post blood meal.

### Southern Hybridization and Diagnostic PCR

Parasite genomic DNA was digested with BamHI and SpeI before transfer to a nylon membrane. The membrane was probed with dioxygenin-labeled exon 5 (DIG High Prime DNA labeling and detection kit, Roche Applied Sciences). DNA hybridization was visualized using a chemiluminescent substrate, following the manufacturer’s instructions. The PbCDPK1 genomic locus in CDPK1 cKO erythrocytic stages and sporozoites as queried using primers p1 and p2 5′CATGACTAGCCCATATAAT and 5′GAAAGTTGGGAATATCCTG.

### RT-PCR

Total RNA extracted from CDPK1- and WT erythrocytic stage parasites (Qiagen RNeasy kit, Qiagen) was reverse transcribed (RETROscript kit, Ambion). PbCDPK1 mRNA was amplified with 5′AGCTTTAAATAGTAGATGGA and 5′ACTTCCTTCAAAATGTTGTC. GAPDH mRNA was amplified with 5′ATGGCAATAA-CAAAAGTCGG and 5′CCCCATGGAATTTGAGCT.

### Infection of HepG2 Cells and Merosome Assay

Sporozoites obtained at day 18–21, post-bloodmeal were added to HepG2 cells cultured on collagen-coated coverslips. Cells were fixed at 40 hours or 65 h post-infection (p.i) with 4% paraformaldehyde followed by permeabilization with cold methanol. Infected cells were identified using immunostaining with an anti-Hsp70 LS mAb [27]. Merosomes were obtained from infected cultures by collecting the media at 66–70 h post-infection and counted in a hemocytometer.

### In vivo Infection

Swiss-webster mice were injected intravenously with CDPK1 cKO or FlpL/TRAP sporozoites (5000 sporozoites/mouse) or erythrocytic stage parasites from CDPK1- or WT (1000 parasites/mouse). Parasitemia was determined daily through microscopic examination of Giemsa stained thin smears.

## References

[1] Singh S, Alam MM, Pal-Bhowmick I, Brzostowski JA, Chitnis CE (2010) Distinct external signals trigger sequential release of apical organelles during erythrocyte invasion by malaria parasites. PLoS Pathog 6: e1000746. 20140184 10.1371/journal.ppat.1000746 PMC2816683

[2] Carruthers VB, Giddings OK, Sibley LD (1999) Secretion of micronemal proteins is associated with toxoplasma invasion of host cells. Cell Microbiol 1: 225–235. 11207555 10.1046/j.1462-5822.1999.00023.x

[3] Carruthers VB, Moreno SN, Sibley LD (1999) Ethanol and acetaldehyde elevate intracellular [Ca2+] and stimulate microneme discharge in Toxoplasma gondii. Biochem J 342 (Pt 2): 379–386. PMC1220475 10455025

[4] Carruthers VB, Sibley LD (1999) Mobilization of intracellular calcium stimulates microneme discharge in Toxoplasma gondii. Mol Microbiol 31: 421–428. 10027960 10.1046/j.1365-2958.1999.01174.x

[5] Chen XM, O’Hara SP, Huang BQ, Nelson JB, Lin JJ, et al (2004) Apical organelle discharge by Cryptosporidium parvum is temperature, cytoskeleton, and intracellular calcium dependent and required for host cell invasion. Infect Immun 72: 6806–6816. 15557601 10.1128/IAI.72.12.6806-6816.2004 PMC529161

[6] Wiersma HI, Galuska SE, Tomley FM, Sibley LD, Liberator PA, et al (2004) A role for coccidian cGMP-dependent protein kinase in motility and invasion. Int J Parasitol 34: 369–380. 15003497 10.1016/j.ijpara.2003.11.019

[7] Agarwal S, Singh MK, Garg S, Chitnis CE, Singh S (2012) Ca(2+) -mediated exocytosis of subtilisin-like protease 1: a key step in egress of Plasmodium falciparum merozoites. Cell Microbiol. 10.1111/cmi.12086 23217145

[8] Dvorin JD, Martyn DC, Patel SD, Grimley JS, Collins CR, et al (2010) A plant-like kinase in Plasmodium falciparum regulates parasite egress from erythrocytes. Science 328: 910–912. 20466936 10.1126/science.1188191 PMC3109083

[9] Garg S, Agarwal S, Kumar S, Shams Yazdani S, Chitnis CE, et al (2013) Calcium-dependent permeabilization of erythrocytes by a perforin-like protein during egress of malaria parasites. Nat Commun 4: 1736. 23591903 10.1038/ncomms2725

[10] Holder AA, Mohd Ridzuan MA, Green JL (2012) Calcium dependent protein kinase 1 and calcium fluxes in the malaria parasite. Microbes Infect 14: 825–830. 22584104 10.1016/j.micinf.2012.04.006

[11] Siden-Kiamos I, Ecker A, Nyback S, Louis C, Sinden RE, et al (2006) Plasmodium berghei calcium-dependent protein kinase 3 is required for ookinete gliding motility and mosquito midgut invasion. Mol Microbiol 60: 1355–1363. 16796674 10.1111/j.1365-2958.2006.05189.x PMC1513514

[12] Billker O, Dechamps S, Tewari R, Wenig G, Franke-Fayard B, et al (2004) Calcium and a calcium-dependent protein kinase regulate gamete formation and mosquito transmission in a malaria parasite. Cell 117: 503–514. 15137943 10.1016/s0092-8674(04)00449-0

[13] Ishino T, Orito Y, Chinzei Y, Yuda M (2006) A calcium-dependent protein kinase regulates Plasmodium ookinete access to the midgut epithelial cell. Mol Microbiol 59: 1175–1184. 16430692 10.1111/j.1365-2958.2005.05014.x

[14] Coppi A, Tewari R, Bishop JR, Bennett BL, Lawrence R, et al (2007) Heparan sulfate proteoglycans provide a signal to Plasmodium sporozoites to stop migrating and productively invade host cells. Cell Host Microbe 2: 316–327. 18005753 10.1016/j.chom.2007.10.002 PMC2117360

[15] Billker O, Lourido S, Sibley LD (2009) Calcium-dependent signaling and kinases in apicomplexan parasites. Cell Host Microbe 5: 612–622. 19527888 10.1016/j.chom.2009.05.017 PMC2718762

[16] Sebastian S, Brochet M, Collins MO, Schwach F, Jones ML, et al (2012) A Plasmodium calcium-dependent protein kinase controls zygote development and transmission by translationally activating repressed mRNAs. Cell Host Microbe 12: 9–19. 22817984 10.1016/j.chom.2012.05.014 PMC3414820

[17] Kato N, Sakata T, Breton G, Le Roch KG, Nagle A, et al (2008) Gene expression signatures and small-molecule compounds link a protein kinase to Plasmodium falciparum motility. Nat Chem Biol 4: 347–356. 18454143 10.1038/nchembio.87 PMC11892688

[18] Ridzuan MA, Moon RW, Knuepfer E, Black S, Holder AA, et al (2012) Subcellular location, phosphorylation and assembly into the motor complex of GAP45 during Plasmodium falciparum schizont development. PLoS One 7: e33845. 22479457 10.1371/journal.pone.0033845 PMC3316498

[19] Thomas DC, Ahmed A, Gilberger TW, Sharma P (2012) Regulation of Plasmodium falciparum glideosome associated protein 45 (PfGAP45) phosphorylation. PLoS One 7: e35855. 22558243 10.1371/journal.pone.0035855 PMC3338798

[20] Bansal A, Singh S, More KR, Hans D, Nangalia K, et al (2013) Characterization of Plasmodium falciparum calcium-dependent protein kinase 1 (PfCDPK1) and its role in microneme secretion during erythrocyte invasion. J Biol Chem 288: 1590–1602. 23204525 10.1074/jbc.M112.411934 PMC3548469

[21] Tewari R, Straschil U, Bateman A, Bohme U, Cherevach I, et al (2010) The systematic functional analysis of Plasmodium protein kinases identifies essential regulators of mosquito transmission. Cell Host Microbe 8: 377–387. 20951971 10.1016/j.chom.2010.09.006 PMC2977076

[22] Panchal D, Govindasamy K, Rana A, Bhanot P (2012) Improved Plasmodium berghei lines for conditional mutagenesis. Mol Biochem Parasitol 184: 52–54. 22450301 10.1016/j.molbiopara.2012.03.005

[23] Azevedo MF, Sanders PR, Krejany E, Nie CQ, Fu P, et al (2013) Inhibition of Plasmodium falciparum CDPK1 by conditional expression of its J-domain demonstrates a key role in schizont development. Biochem J 452: 433–441. 23548171 10.1042/BJ20130124

[24] Lourido S, Tang K, Sibley LD (2012) Distinct signalling pathways control Toxoplasma egress and host-cell invasion. EMBO J 31: 4524–4534. 23149386 10.1038/emboj.2012.299 PMC3545288

[25] Garrison E, Treeck M, Ehret E, Butz H, Garbuz T, et al (2012) A forward genetic screen reveals that calcium-dependent protein kinase 3 regulates egress in Toxoplasma. PLoS Pathog 8: e1003049. 23209419 10.1371/journal.ppat.1003049 PMC3510250

[26] McCoy JM, Whitehead L, van Dooren GG, Tonkin CJ (2012) TgCDPK3 regulates calcium-dependent egress of Toxoplasma gondii from host cells. PLoS Pathog 8: e1003066. 23226109 10.1371/journal.ppat.1003066 PMC3514314

[27] Falae A, Combe A, Amaladoss A, Carvalho T, Menard R, et al (2010) Role of Plasmodium berghei cGMP-dependent protein kinase in late liver stage development. J Biol Chem 285: 3282–3288. 19940133 10.1074/jbc.M109.070367 PMC2823412

